# A Novel Regulatory T Cell Population in the Gut

**DOI:** 10.1371/journal.pbio.1001834

**Published:** 2014-04-08

**Authors:** Caitlin Sedwick

**Affiliations:** Freelance Science Writer, San Diego, California, United States of America

When microbes invade, they are taken up by body cells or immune cells such as monocytes, which digest them into protein fragments, or antigens, that are then presented on their cell surface. These antigens are recognized by a specialized receptor on the surface of T cells (the T cell receptor, or TCR), whose signaling prompts secretion of proteins called cytokines that coordinate the immune system's efforts to fight off the pathogen.[Fig pbio-1001834-g001]

**Figure pbio-1001834-g001:**
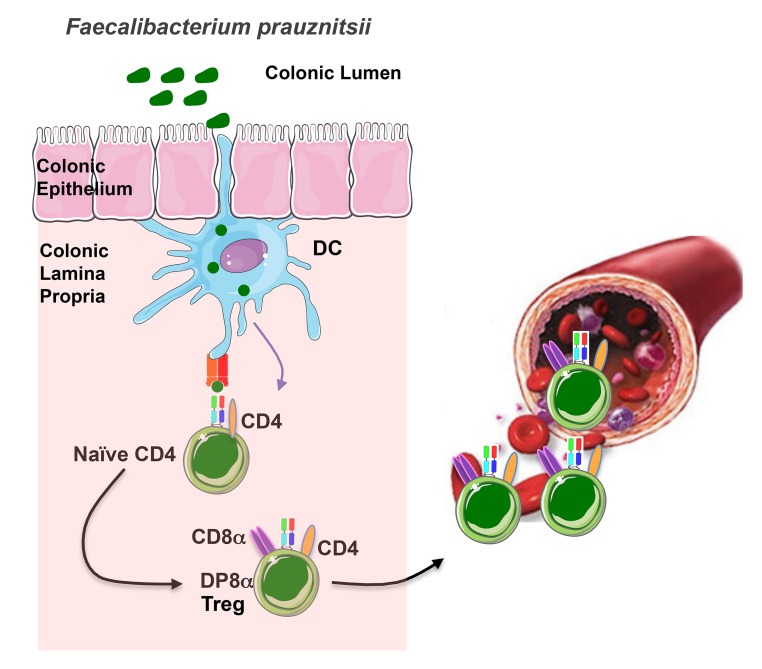
In the colonic mucosa, antigens of *Faecalibacterium prausnitzii* (a clostridium bacteria from the gut microbiota) are presented by dendritic cells. Recognition of these antigens by the CD4 T cells equipped with a specific TCR contributes to their differentiation into FoxP3-lacking regulatory T cells (Treg) characterized by the co-expression of CD4 and CD8α. Most of these Treg stay in the colonic mucosa where they prevent excessive inflammatory responses. A fraction of them migrate into the blood, likely contributing to the immune tolerance outside the gut.

Each individual T cell expresses just one of myriad different versions of the TCR, each of which responds to a particular antigen. This wide diversity allows the recognition of a large repertoire of threats, but also carries the risk of inappropriate reactivity against normal tissues. While T cells that may mount such attacks are usually weeded out during their development, autoreactive cells do occasionally appear. In addition, changes to body homeostasis may sometimes provoke T cell autoimmunity. As a failsafe against these problems, a specialized type of T cell called the “T regulatory” cell (Treg) reins in inappropriate immune responses by secreting immunosuppressive cytokines such as IL-10. Tregs can be distinguished from other types of T cells primarily by their expression of the transcription factor FoxP3, together with a characteristic set of cell surface proteins.

The ability to restrict immune responses is delicately balanced. For example, the immune system normally tolerates several species of commensal gut bacteria, and does not mount immune responses against them. However, perturbances to the mixture of commensal bacteria communities can lead—through mechanisms that remain unclear—to bowel inflammation, and sometimes serious autoimmune diseases such as inflammatory bowel disease (IBD) or ulcerative colitis. In their paper published this month in *PLOS Biology*, Guillaume Sarrabayrouse, Frédéric Altare, Francine Jotereau, and colleagues describe a new type of T cell that mediates immune tolerance, which may be important for preventing bowel inflammation.

While examining the lymphocyte population in the lamina propria of normal human gut mucosa, Sarrabayrouse et al. noticed a group of T cells that simultaneously expressed two different types of T cell–specific surface proteins, CD4 and CD8αα. The researchers were intrigued by these double-positive CD4CD8αα lamina propria lymphocytes (DP8α LPL) because most T cells outside the thymus (where developing T cells reside) express only one of either CD4 or CD8. The authors decided to investigate the function of these cells and found that, compared with CD4 single-positive T cells derived from the same site, DP8α LPL expressed surface markers classically associated with Treg. Curiously, however, DP8α LPL did not express FoxP3. Despite their lack of FoxP3, DP8α LPL secreted a high amount of IL-10 and they were potently immunosuppressive, able to prevent the maturation and proliferation of other types of immune cells in vitro.

Previous studies had indicated that certain commensal bacteria, particularly those of the *Clostridium* IV group, can induce the appearance of FoxP3 regulatory T cells in mice. The authors therefore studied whether DP8α LPL responded to these commensals. In fact, although DP8α LPL expressed a widely heterogeneic population of TCRs, when presented with human monocytes pre-incubated with a *Clostridium* IV group bacterium, *Faecalibacterium prausnitzii* (*F prau*), a large portion of DP8α LPL responded by proliferating and secreting cytokines. Other commensals, even closely related bacteria of the *Clostridium* IV group, failed to elicit any response from DP8α LPL.

Occasionally, bacteria can activate T cells nonspecifically by secreting proteins called “superantigens,” which activate TCR signaling regardless of whether pathogen-derived antigens are being presented to the T cell. But Sarrabayrouse et al. showed that DP8α LPL proliferative and cytokine responses to *F prau* did not depend on superantigens, and that DP8α LPL were specifically reactive to *F prau*–derived antigens.

Further studies showed that DP8α T cells could be detected not only in the gut but also in the peripheral blood (such cells were dubbed DP8α PBL). DP8α PBL differed from their LPL counterparts in that they lacked expression of cell surface markers associated with regulatory T cells, and a lower fraction of DP8α PBL displayed reactivity to *F prau*. The authors suspect DP8α PBL may be derived from DP8α LPL, although what role they play in the body remains unclear.

Sarrabayrouse et al. next investigated what happens to DP8α T cells during gut inflammation. They observed that both DP8α LPL and PBL populations were smaller in patients with active IBD than in healthy donors, which correlates with the fact that *F prau* levels drop precipitously during IBD flare-ups. Furthermore, the authors observed that DP8α PBL and LPL from IBD patients sometimes failed to proliferate or secrete cytokines in response to *F prau*. Collectively, these data suggest that DP8α T cells are a novel type of regulatory T cell that respond specifically to *F prau*, and whose loss or dysfunction may affect immune homeostasis in the gut.

**Sarrabayrouse G, Bossard C, Chauvin J-M, Jarry A, Meurette G, et al. (2014) CD4CD8αα Lymphocytes, a Novel Human Regulatory T Cell Subset Induced by Colonic Bacteria and Deficient in Inflammatory Bowel Disease Patients.** doi:10.1371/journal.pbio.1001833

